# Perspectives, practices, and challenges of online teaching during COVID-19 pandemic: A multinational survey

**DOI:** 10.1016/j.heliyon.2023.e19102

**Published:** 2023-08-11

**Authors:** Jaber S. Alqahtani, Renata G. Mendes, Maria Isabel Triches, Tatiana de Oliveira Sato, Jithin K. Sreedharan, Abdulelah M. Aldhahir, Abdullah A. Alqarni, Reynie Purnama Raya, Mohammed Alkhathami, Arulanantham Zechariah Jebakumar, Ayadh Yahya AlAyadi, Abdullah S. Alsulayyim, Abdullah S. Alqahtani, Saeed M. Alghamdi, Ibrahim A. AlDraiwiesh, Musallam Alnasser, Rayan A. Siraj, Abdallah Y. Naser, Hassan Alwafi, Saad M. AlRabeeah, Mohammed D. AlAhmari, Ami Kamila, Heba Bintalib, Eman M. Alzahrani, Tope Oyelade

**Affiliations:** a Department of Respiratory Care, Prince Sultan Military College of Health Sciences, Dammam, Saudi Arabia; b Cardiopulmonary Physiotherapy Laboratory, Department of Physical Therapy, Federal University of São Carlos, SP, Brazil; c Respiratory Therapy Department, Faculty of Applied Medical Sciences, Jazan University, Jazan 45142, Saudi Arabia; d Department of Respiratory Therapy, Faculty of Medical Rehabilitation Sciences, King Abdulaziz University, Jeddah, Saudi Arabia; e Institute for Global Health, Faculty of Population Health Sciences, University College London, London NW3 2PF, UK; f Faculty of Science, Universitas ‘Aisyiyah Bandung, Bandung 40264, Indonesia; g Vice Deanship of Post Graduate Studies and Research, Prince Sultan Military College of Health Science, Dammam, Saudi Arabia; h Respiratory Care Program, College of Applied Medical Sciences, Umm Al-Qura University, Makkah 24382, Saudi Arabia; i Respiratory Therapy Department, King Faisal University, Al-Ahsa, 31982, Saudi Arabia; j Department of Applied Pharmaceutical Sciences and Clinical Pharmacy, Faculty of Pharmacy, Isra University, Amman 11622, Jordan (AN); k Faculty of Medicine, Umm Al Qura University, 21514, Mecca, Saudi Arabia (HA); l Department of Public Health, Faculty of Medicine, Public Health and Nursing, Universitas Gadjah Mada, Yogyakarta, Indonesia; m UCL Respiratory, University College London, London, UK; n Department of Respiratory Care, King Saud bin Abdulaziz University for Health Sciences, Jeddah, Saudi Arabia; o King Abdullah International Medical Research Center, Jeddah, Saudi Arabia; p Curriculum & Study Plan Unit, Vice Deanship of Academic Affairs, Prince Sultan Military College of Health Sciences, Dammam, Saudi Arabia; q Institute for Liver and Digestive Health, Division of Medicine, University College London, London, UK

**Keywords:** COVID-19, Online teaching, Pedagogy, Perception, Digital learning

## Abstract

The result of the movement restrictions during the COVID-19 pandemic was an impromptu and abrupt switch from in-person to online teaching. Most focus has been on the perception and experience of students during the process. The aim of this international survey is to assess staffs' perspectives and challenges of online teaching during the COVID-19 lockdown. Cross-sectional research using a validated online survey was carried out in seven countries (Brazil, Saudi Arabia, Jordan, Indonesia, India, the United Kingdom, and Egypt) between the months of December 2021 and August 2022, to explore the status of online teaching among faculty members during the COVID-19 pandemic. Variables and response are presented as percentages while logistic regression was used to assess the factors that predict levels of satisfaction and the challenges associated with online instruction. A total of 721 response were received from mainly male (53%) staffs. Most respondents are from Brazil (59%), hold a Doctorate degree (70%) and have over 10 years of working experience (62%). Although, 67% and 79% have relevant tools and received training for online teaching respectively, 44% report that online teaching required more preparation time than face-to-face. Although 41% of respondents were uncertain about the outcome of online teaching, 49% were satisfied with the process. Also, poor internet bandwidth (51%), inability to track students' engagement (18%) and Lack of technical skills (11.5%) were the three main observed limitations. Having little or no prior experience of online teaching before the COVID-19 pandemic [OR, 1.58 (95% CI, 1.35–1.85)], and not supporting the move to online teaching mode [OR, 0.56 (95% CI,0.48–0.64)] were two main factors independently linked with dissatisfaction with online teaching. While staffs who support the move to online teaching were twice likely to report no barriers [OR, 2.15 (95% CI, 1.61–2.86)]. Although, relevant tools and training were provided to support the move to online teaching during COVID-19 lockdown, barriers such as poor internet bandwidth, inability to track students’ engagement and lack of technical skills were main limitations observed internationally by teaching staffs. Addressing these barriers should be the focus of higher education institution in preparation for future disruptions to traditional teaching modes.

## 1 Lay Abstract

COVID-19-related restrictions resulted in global lockdown of essential facilities including higher education institutions. This resulted in disruption to the face-to-face teaching. We conducted a cross-sectional survey of staff members in higher education around the globe to evaluate their perception of the abrupt transfer to online teaching during the COVID-19 lockdowns. Poor internet bandwidth, inability to track students’ engagement and lack of technical skills were a global barrier to online teaching at the peak of the COVID-19 pandemic.

## 2 Introduction

In 2019, the coronavirus disease (COVID-19) was identified in Wuhan, China and declared as a pandemic by the World Health Organization (WHO) in March 2020 [1]. Because of implementing the WHO regulatory guidance, people's lifestyles have tremendously changed in almost all aspects of their daily routine. On the other hand, the pandemic exposed flaws in most countries' health, economic and educational systems' readiness for a worldwide catastrophe [2].

The educational institutions undertook the necessary precautionary steps, including displacement of students from on-campus teaching, reduction of staff attending on-site and entire transition of classes from in-person to online education. The United Nations (UN) emphasized that a COVID-19 pandemic is considered the most extraordinary historic disruption of the education regime worldwide [3]. More than nearly 1.6 billion students in 190 countries were affected [3]. These sudden and unprecedented changes within a limited time mandated the restructuring of pedagogies, including delivery routes of the course contents, adopting a new strategy to assess students’ academic achievements, adapting existing course material to be taught online, and ensuring the appropriateness of technology to deliver the contents of the course and interact with students effectively [4]. Despite these challenges, the higher education systems were perceived to have an excellent pre-existing online infrastructure for teaching, learning and communication in most countries to tackle these challenges [5,6].

Nevertheless, the sudden transition from face-to-face to online teaching (OT) aroused concerns regarding the teaching faculty's capability to effectively utilize the technology to teach and assess the students' academic performance, and the level of technical support they get from their educational institutions [7,8]. As a result, multitudes of studies were published on OT transition due to COVID-19 to explore the teaching faculty experiences on OT. Jonathan et al. presented a single center experience in USA of faculty and trainees' perceptions to compare in-person learning versus OT during COVID-19 [9]. The researchers found that over half of faculty and trainees perceived in-person learning more positively than OT on most items assessed. The items rated higher with OT included attention during lectures, the interaction between trainees and faculty teaching, overall enjoyment, and participation in discussions. Furthermore, a survey of Egyptian physical therapy educators at Egyptian universities revealed that the most (83%) challenging factor experienced during OT is the lack of OT experience [10]. Only (22.9%) of educators had positive attitudes toward OT, less than half of them (47.6%) were satisfied with OT experience, and (9%) of them perceived the beneficial quintessence of OT for physical therapy colleges [10]. Interestingly, (91%) of educators have a negative attitude toward the preparedness, resources, and infrastructure of their institutes for the implementation of OT. Moreover, in France, (65%) of the surveyed pediatric instructors revealed that they did not obtain proper quality training as anticipated [11]. In United Arabian Emirates (UAE), medical and health sciences colleges were surveyed to measure the faculty and students' satisfaction with OT during the pandemic [12]. The overall faculty satisfaction was 74.3% and the time required to prepare assessment and teaching materials, increased workload, technical issues and enhancing engagement were the main factors that impeded their level of satisfaction. According to research conducted by Alqahtani et al., 2022 in Saudi Arabia [13], the vast majority of academics had a favorable impression of online education (62%), with 71% reporting high levels of satisfaction with this mode of instruction. Twenty-five percent of those who participated in the survey noted that slow internet was an issue, and another 20% said they couldn't monitor student participation [13].

These studies provided valuable insights into our present understanding of OT due to COVID-19. Simultaneously, there are some limitations in the current literature concerning this research topic after conducting a comprehensive search. These limitations include most of the studies recruited a small number of teaching faculty in their analysis, single center experiences, all the studies linked to specific geographical areas or certain disciplines, and the existed studies provided a snapshot of faculty's experience in a certain period of the pandemic rather than a longitudinal experience. Therefore, to broaden the scope of previous studies and address the gap in the current literature, the study was conducted to investigate multinational teaching faculty perceptions, attitudes, challenges, and satisfaction with online teaching during the covid-19 pandemic. The data might generate empirical evidence to help future OT developments be more inclusive.

## 3 Methods

⮚ **Instrumental Questionnaire**

A cross-sectional survey was conducted online via (Survey Monkey) between December 5th, 2021 and August 5th, 2022. The questionnaire was divided into four parts: background information, past exposure to online teaching, existing methods, and resources, as well as anticipated outcomes, levels of satisfaction, and potential barriers to implementation during COVID-19. Based on recent literature [13], the questions were framed to capture the perception and challenges of online teaching by educators by collaboration between a panel of education professionals made up of the authors. The survey's content and face validity were then assessed using a sample of randomly selected educators with over 10 years of experience in higher education teaching across the field of natural and medical sciences spread across the various study locations. Thus, the final draft of the questionnaire incorporated feedbacks from these professionals followed by comprehensive translation into the various languages of the countries of study. Using the Cronbach's alpha reliability coefficient, we found that the instrument's internal consistency was 0.84.

The final questionnaire was divided into various sections, each with its own set of multiple-choice questions. In the first section, participants were provided with a comprehensive description of the study and provided with contact details for enquiry. They were then asked to consent to anonymous participation in the survey and only have access to the questions once consent has been given. The second section assesses participants’ background information, including their location, gender, age, and level of education, area of expertise, and number of years in the field, student body size, and kind of school. The second section also includes questions on online education prior to the COVID-19 outbreak, including the participants' perspectives and experiences. Further, participants were asked 13 questions on the state of online education at the time of the survey. The concluding part of the section consisted of three questions on anticipations, experiences, and challenges of online teachings during COVID-19. Details of the survey questions is provided as a supplemental material.

⮚ **Study population**

Research participants were recruited from a variety of fields using a convenience sampling method. All the main disciplines taught at universities and colleges in these countries (Brazil, Saudi Arabia, Jordan, Indonesia, India, United Kingdom, and Egypt) were eligible and included. We wanted to get this survey into the hands of as many teachers and professors as possible, so we promoted it on social media and via our professional networks (Twitter, LinkedIn, and WhatsApp). The main distribution channel of the survey is via direct mailing list (emails) and during staffs’ networking, social or academic events. Inclusion criteria include being a teaching faculty member in a higher education institution with more than 6 months of experience prior to the COVID-19 lockdown and the move to online teaching. The Institutional Review Board of Prince Sultan Military College of Health Sciences approved this study, reference number (IRB-2022-RC-004) as well as the UFSCar Ethics Committee approved it (CAAE: 56151622.5.0000.5504). All respondents gave their informed consent prior to taking the survey. No power calculation was set as this study was an exploratory and not required a formal analysis.

⮚ **Statistical Analysis**

Data was analyzed using the Statistical Package for the Social Sciences (SPSS software, Version 28). Specifically, variables were presented as percentages. While logistic regression analysis was performed to identify variables associated with degree of satisfaction as well as determinants of perceived barriers to online education among faculty members. A test was deemed statistically significant if the two-tailed p value < 0.05.

## 4 Results

⮚ **Demographic characteristics**

This study involves 721 participants from 8 countries around the world. Majority of respondents are male 379 (53%). Brazil has the highest response (59%) followed by Saudi Arabia (17%) and Jordan (10%). Also, participants between the age 31–41 (29%) and 41–50 (28%) years represents the highest proportion. Whereas those aged 21–31 (9%) and over 60 years (13%) were the least represented. Respondents with student cohort size of 21–40 (39%) and 41–60 (20%) were mostly represented, while 81–100 depicts the lowest percentage (6%). Indeed, only 12% of respondents cater to a very large cohort size of more than 100 students. In terms of the academic qualifications of respondents, most of the respondents (70%) hold a Doctor of Philosophy (PhD), followed by Masters (30%), Bachelor (5%) degree and then diploma (0.1%). Humanities and Social Sciences have the largest proportion in terms of specialization (36%), while Biological Sciences show the smallest proportion (2.4%). Majority of the respondents have more than 10 years of working experience (62%), a significant proportion of which work in government\public institutions (64%; Table 1).

⮚ **Practice and perceptions regarding online teaching before COVID-19**

**Table 1:** Demographic data and characteristics of the respondents (n = 721).

Variable	N (%)
Gender	
Male	379 (53)
Female	342 (47)
Age Groups	
21–30	62 (8.6)
31–40	209 (29.0)
41–50	202 (28.0)
51–60	155 (21.5)
>60	92 (12.8)
Size of the student cohort	
1–20	112 (15.6)
21–40	283 (39.4)
41–60	140 (19.5)
61–80	51 (7.1)
81–100	43 (6.0)
>100	92(12.4)
Academic Qualification	
Diploma	1 (0.1)
Bachelors	33 (4.6)
Masters	179 (24.9)
PhD	507 (70.4)
Area of Specialization	
Humanities and social sciences	258 (35.8)
Professions and Applied Sciences	200 (27.7)
Formal sciences	146 (20.2)
Natural sciences	117 (16.2)
Years of teaching experience	
<10	272 (37.7)
>10	449 (62.3)
Type of institution working in	
Private	250 (35.0)
Public/Government	471 (65.0)
What is your Country of Residence	
Brazil	423 (58.7)
Saudi Arabia	122 (16.9)
Jordan	74 (10.3)
Indonesia	29 (4.0)
India	27 (3.7)
United Kingdom	26 (3.6)
Egypt	20 (2.8)

Before COVID-19 pandemic, only 26% and 18% of participants frequently or occasionally utilized online teaching, while 22% and 34% rarely or never use online teaching methods respectively. In addition, almost 30% of the respondents believe that the moving of teaching to an online theme is easy to apply whereas, 51% have different views that will be a difficult process, still, and there are about 15% show a neutral position about online teaching. Moreover, from the perspective of the participants about supporting the switch to online teaching, about 64% of the participants were supportive. Furthermore, about 21% strongly did not support the move to online teaching mode during COVID-19 pandemic while 15% of respondents were neutral (Table 2).

⮚ **Preparedness and tools for online teaching during COVID-19**

**Table 2:** Experience and perceptions regarding online teaching before COVID-19.

Variable	N (%)
How often did you use online/remote teaching before COVID-19?	
Frequently used	190 (26.4)
Occasionally used	126 (17.5)
Rarely used	159 (22.1)
Never used	245(34.0)
What is your overall perception of moving teaching online?	
Very easy	59 (8.2)
Easy	158 (21.9)
Somewhat difficult	301 (41.70)
Very Difficult	67 (9.3)
How willing were you to support moving teaching online?	
Strongly support	234 (32.5)
Somewhat support	227 (31.5)
Somewhat opposed	98 (13.6)
Strongly opposed	55 (7.6)

In terms of how institutions were prepared for online teaching during the COVID-19 lockdown, 67% of respondents confirmed that relevant tools for delivering online teachings were provided, 11% of the did not have relevant tools needed. Also, 79% of respondents received some form of training for online teaching which were mainly provide via live broadcasts (58%) and online courses (26%). In terms of the tools that were mostly utilized for online teaching during COVID-19 lockdowns, Blackboard (32%) and Google Meet (32%) were the most popular with respondents, with Microsoft Team being the least utilized (7%). Indeed, Blackboard (44%) was also the main platform for teaching material distribution followed by a combination of Google Drive/Google Classroom, Emails and Online Blackboard (17%). Prior to teaching sessions, 61% of respondents distributed teaching materials in less than one week while 33% did this more than one week before. In terms of preparation time for online teaching, 83% spent up to 4 h 17% spent more than 4 h. Further, 44% respondent show that online class takes more time than offline class while the rest (56%) believes similar or lesser time was required in preparing for online sessions. Overall, the most popular tool for assessment/evaluation was a combination of assignment, quiz and examination (59%) mostly using Google Forms (40%). For respondents whose course includes practical skills teaching (53%), practical teaching materials were mostly delivered by Post (47%) or collected from campus (43%), with evaluation of practical skills performed mostly through live presentations (34%) or written reports (31%; Table 3)

⮚ **Expectations, satisfaction, and barriers of online teaching during COVID-19**

**Table 3:** **Current practices and tools of online teaching during COVID-19**.

Variable	N (%)
Were relevant tools (software, hardware etc.) provided for online teaching?	
Yes	480 (66.7)
No	81 (11.3)
**Was adequate training provided for online teaching?**	
Yes	343 (47.7)
No	156 (21.6)
**How was training provided?**	
Online course	184 (25.5)
Self-Training	26 (3.6)
Live broadcasts	420 (58.3)
Online course & One-on-one sessions	24 (3.3)
No training was provided	31 (4.3)
**Which online teaching tools do you mostly use for online teaching?**	
Blackboard	230 (31.9)
Blackboard and zoom	80 (11.1)
Zoom only	133 (18.4)
Microsoft Teams	50 (6.9)
Google Meet	228 (31.6)
**On average, how much time you spend on preparing for a session of online class?**	
<2 h	250 (34.7)
2–4 h	347 (48.2)
>4 h	123 (17.1)
**Time you spend to prepare a session of an online class is:**	
The same time spend for a session of offline class	284 (39.4)
Less than time spend for a session of offline class	119 (16.5)
More than spend for a session of offline class	317 (44.0)
**How do you distribute your teaching material?**	
Blackboard	282 (44.2)
Combination of Microsoft Teams and Email	30 (4.7)
Combination of Blackboard and Email	61 (9.6)
Google Drive/Google Classroom, Emails, Online Blackboard (e.g., Moodle)	105 (16.5)
Microsoft Teams	60 (9.4)
Google Drive/Google Classroom, Emails, WhatsApp	7 (1.1)
Google Drive/Google Classroom	93 (14.6)
**When do you usually distribute teaching materials?**	
Less than a week before class	444 (62.3)
More than a week before class	227 (31.8)
During or after class	25 (3.5)
It varies	17 (2.4)
**How did you conduct evaluation for your online class?**	
Assignment, Quiz & Examination	423 (58.8)
Assignment & Quiz	86 (11.9)
Assignment & Examination	153 (21.3)
Quiz	23 (3.2)
Assignment	18 (2.5)
All the above	17 (2.4)
**What instrument do you use for evaluation?**	
Blackboard	51 (7.2)
Google forms	280 (39.8)
Kahoot	16 (2.3)
Google Forms, Kahoot	20 (2.8)
Google forms, Moodle	5 (0.7)
Moodle	92 (13.1)
Zoom pooling	77 (10.9)
Others	163 (23.1)
**Does your course include practical skills teaching?**	
Yes	379 (52.6)
No	342 (47.4)
**For practical skill materials, how do you distribute them during the COVID-19 lockdown?**	
Arrange time for Collected from Campus	21 (42.9)
Blackboard	5 (10.2)
Sent by post	23 (46.9)
**How do you conduct evaluation for practical skill?**	
Live and Recorded presentation	61 (17.9)
Live student presentation	117 (34.3)
Reports	107 (31.4)
Recorded student presentation	36 (10.6)
Others	20 (5.9)

The expectations of respondents about moving to online teaching during COVID-19 lockdowns were mostly positive (43%) or uncertain (41%). Conversely, once teachings moved online, most were satisfied with the mode of teaching (49%) while 30% of respondents were satisfied. Regarding the limitations, 51% of the respondents cited poor internet strength (bandwidth) as the main barrier to effective online teaching during COVID-19 lockdown followed by the inability to track students' engagement (18%). Lack of technical skills was also reported as a main barrier with 11.5% of respondents (Table 4).

⮚ **Predictors for satisfaction and barriers of online teaching during COVID-19**

**Table 4:** Expectations, satisfaction, and barriers of online teaching during COVID-19.

Variable	N (%)
What was your general expectations of moving to online teaching during COVID-19?	
Positive	309 (42.9)
Negative	114 (15.8)
**What is your overall satisfaction of online education?**	
Very dissatisfied	66 (9.2)
Dissatisfied	149 (20.7)
Satisfied	312 (43.3)
Very satisfied	41 (5.7)
**What were the main barriers of online teaching based on your experience?**	
Poor Internet	368 (51.0)
Inability to track students' engagement	132 (18.3)
Lack of technical skills	83 (11.5)
No barriers	48 (6.7)
Lack of training	32 (4.4)
Creating online teaching contents	17 (2.4)
Lack of adequate hardware	15 (2.1)
Childcare and family responsibilities	14 (1.9)
Long teaching time	10 (1.4)
Lack of adequate software	2 (0.3)

Logistic regression was used to analyze the relationship between the level of satisfaction and the independent variables such as years of experience, age, gender, size of the cohort, nationality, perception, and support of moving teaching online. Having little or no prior experience of online teaching before the COVID-19 pandemic [OR, 1.58 (95%CI, 1.35–1.85)], and not supporting the move to online teaching mode [OR, 0.56 (95% CI, 0.48–0.64)] are two main factors independently linked with dissatisfaction with online teaching. Conversely, prior support for online teaching is also independently associated with no barriers [OR, 2.14 (95%CI, 1.60–2.86); Table 5].

**Table 5:** Multivariable logistic regression analysis of predictors for satisfaction and barriers.

Variable	Odds ratio (OR)	95% Confidence Interval for odds ratio		P-value
		Lower Bound	Upper Bound	
Level of Satisfaction				
Years of Teaching Experience	1.09	0.85	1.39	0.48
Age group	0.96	0.79	1.16	0.70
Gender	0.93	0.67	1.30	0.70
What is the size of the student cohort you currently support?	0.93	0.84	1.04	0.23
Nationality	0.91	0.83	1.00	0.05
Frequent user of online/remote teaching prior to COVID-19	1.58	1.35	1.85	**<0.001**
Lack of support for moving teaching online	0.56	0.48	0.64	**<0.001**
**Barriers (from multiple barriers to no barrier)**				
Years of Teaching Experience	1.13	0.73	1.75	0.57
Age group	0.75	0.52	1.08	0.12
Gender	0.79	0.41	1.51	0.48
Size of the student cohort	1.01	0.83	1.24	0.86
Nationality	0.97	0.81	1.15	0.72
Frequent user of online/remote teaching prior to COVID-19	1.25	0.95	1.65	0.10
Support moving teaching online	2.14	1.60	2.864	**<0.001**

## 5 Discussion

In this international survey of higher education institution teaching staffs, we investigated the general perception and barriers to the switch to online teaching during the global COVID-19 restrictions. Our findings showed that the general perception of online teaching due to COVID19 pandemic was mostly positive among teaching staffs who also report been provided with relevant equipment and technology as well as training for online teaching. Further, most teaching staffs surveyed also supported the move to online teaching under global COVID-19 restrictions with mostly positive expectations and satisfaction level. However, barriers such as poor internet bandwidth and the inability to track students’ engagement were highlighted.

The COVID-19 pandemic resulted in restrictions to general movement and closure of schools, including higher education institutions around the world [7]. The consequence is an abrupt switch to online teaching to ensure continuation of education albeit, with little to no time to carefully plan [14]. Previous studies have examined the satisfaction of students and staffs with online teaching mode due to COVID-19 lockdowns with various but similar findings [[15], [16], [17]]. For instance, in a survey of 280 students and 50 faculty students, significant number of responders highlighted the usefulness of online education during the pandemic [18]. Also, the use of online platform for the delivery of histology course was reported by Darici et al. to be effective and well received with positive feedback by medical anatomy students during COVID-19 pandemic [19]. Put together, the move to online mode of teaching during COVID-19 pandemic and consequent lockdowns was generally perceived to be necessary by students and staffs globally.

In terms of limitations and barriers, this and previous studies show that the problem of poor internet bandwidth is universal. Indeed, Dost et al. surveying over 2700 medical students from the United Kingdom reported that aside family distraction, poor internet connection was the main reported barrier to smooth online learning [20]. Similarly, study by Almahasees et al. of Jordanian university students and staffs reported poor internet as a main limitation [18]. These works have been supported by other studies from India [21,22], Russia [23], and Romania [24] amongst others. Another major limitation reported in this survey is the inability to track students’ performance or understanding. This barrier is also reported by a previous study conducted in Pakistan [25]. Although, the benefit of online learning such as the more relaxed learning environment as well as the ability to learn at pace have been previously highlighted [26], the intrinsic barrier to online learning is highlighted in fields were human contact or practical skills are essential. This include medical training, biomedical sciences amongst others for which alternative to tactile learning is not currently available. Indeed, this limitation was highlighted in the study of ninety-eight medical students on gynecology and obstetrics course by Olmes et al. which requires skill set only available through real-life, face-to-face practice [27].

Result of multivariate logistic regression analysis to evaluate the factors that may predict the tendency to be satisfied with online teaching or experience barriers showed that staffs with previous experience of online teaching as well as those that supported the movement of all teaching online were more likely to be relatively satisfied. Also, staffs that supported online teaching mode have a lower propensity to report barriers. Conversely, this may be interpreted as follows. Intuitively, staffs with frequent previous use of online teaching modes have better experience with the online teaching tools and are more trained. This consequently mean that their use of online teaching platforms is less phased with barriers resulting in a better teaching experience. Also, this group are more likely to have the relevant resources and tools for delivery of online class as well as a better understand of the pedagogic dynamics of online teaching delivery. Further, support for the switch to online mode may be based on the perceived need to protect loved ones or the “fear of COVID” which may be provide a balance against any perceived barriers. Indeed, the effect of “fear of COVID” on preventative behavior is well documented [[28], [29], [30], [31]].

This study has limitations. Firstly, because of the retrospective nature of the survey, there is a problem of recollection error which may result in potential bias. However, this is a general limitation of studies that depends on self-reported data. Also, Africa, a continent with unique sets of teaching challenges, is not represented in the dataset and some of the included countries have significantly low respondent. Thus, the result may not be generalizable to countries from these affected regions. Thirdly considering that this is a multinational study, a larger and more uniform representative samples size may provide a stronger result with higher significance. However, this is the first survey conducted across countries and continent assessing the perception and barriers to moving teaching online as experienced by teaching staff members. Future studies could consider this limitation for a better understanding of the factors that limits the efficiency of online teaching.

## 6 Conclusion

Although staffs were positive about and well prepared for the switch to online teaching during COVID-19 pandemic, barriers such as poor internet speed and inability to perceive student understanding, limits the quality of teaching delivery. Also, previous frequent use of online teaching platform is a driving factors for positive staffs’ experience. Policy makers should consider implementing plans that allow for increased exposure to online teaching platform in preparation for future disruption to traditional teaching modes. This may be in the form of increased training and continuous professional development that focuses on improving knowledge of online teaching technologies and tools. Because online teaching has unique pedagogic challenges that differs from traditional teaching techniques exploring innovative methods such as virtual reality, artificial intelligence, machine learning, gamifications and other techniques could be introduced to enhance remote teaching in future.

## Declarations

### Author contribution statement

Tope Oyelade; Jaber S Alqahtani: Conceived and designed the experiments; Performed the experiments; Analyzed and interpreted the data; Contributed reagents, materials, analysis tools or data; Wrote the paper.

Renata G Mendes; Maria Isabel Triches; Tatiana de Oliveira Sato; Jithin K Sreedharan; Abdulelah M Aldhahir; Abdullah A Alqarni; Reynie Raya Purnama; Mohammed Alkhathami; Arulanantham; Zechariah Jebakumar; Ayadh Yahya AlAyadi; Abdullah S Alqahtani; Saeed M Alghamdi; Ibrahim A AlDraiwiesh; Musallam Alnasser; Rayan A Siraj; Abdallah Y Naser; Hassan Alwafi; Saad M AlRabeeah; Mohammed D AlAhmari; Ami Kamila; Heba Bintalib; Eman M Alzahrani: Performed the experiments; Contributed reagents, materials, analysis tools or data; Wrote the paper.

### Data availability statement

Data will be made available on request.

### Additional information

No additional information is available for this paper.

## Declaration of competing interest

The authors declare that they have no known competing financial interests or personal relationships that could have appeared to influence the work reported in this paper.
